# Harnessing Extracellular Vesicles for Targeted Drug Delivery in Ovarian Cancer

**DOI:** 10.3390/pharmaceutics17040528

**Published:** 2025-04-17

**Authors:** Jang-Hyuk Yun, Yoo Rim Noh, Seongkyeong Yoo, Soohyun Park, Yunsup Choi, Jiyeon An, Iljin Kim

**Affiliations:** 1 College of Veterinary Medicine and Institute of Veterinary Science, Kangwon National University, Chuncheon 24341, Republic of Korea; 2 Department of Pharmacology and Program in Biomedical Science and Engineering, Inha University College of Medicine, Incheon 22212, Republic of Korea; 3 Research Center for Controlling Intercellular Communication, Inha University College of Medicine, Incheon 22212, Republic of Korea

**Keywords:** extracellular vesicles, ovarian cancer, drug delivery, targeted therapy, precision medicine

## Abstract

Ovarian cancer remains one of the most lethal gynecologic malignancies, primarily due to late-stage diagnosis, high recurrence rates, and the development of chemoresistance. Although targeted therapies have improved patient outcomes, their efficacy is often limited by off-target toxicity and acquired drug resistance. Extracellular vesicles (EVs), nanoscale vesicles naturally released by cells, have emerged as promising carriers for precision drug delivery. This review provides a comprehensive overview of recent advances in EV-based therapeutic strategies for ovarian cancer, including the delivery of chemotherapeutic agents, nucleic acid therapeutics, and immunomodulatory molecules. We further explore innovative engineering approaches to enhance targeting specificity, such as surface modification, cell source selection, biomaterial integration, and magnetic nanoparticle-assisted delivery. Key translational challenges in bringing EV-based therapies to clinical application are also addressed. Collectively, these insights underscore the transformative potential of EV-based platforms in advancing targeted and personalized treatment for ovarian cancer.

## 1. Introduction

Ovarian cancer is the fifth leading cause of cancer-related deaths among women, with high-grade serous ovarian carcinoma (HGSOC) representing the most aggressive and prevalent subtype. Despite advancements in surgical techniques and chemotherapy, the recurrence and emergence of chemoresistance remain major clinical challenges [1]. Over the past decade, targeted therapies, including monoclonal antibodies and small molecules, have improved clinical outcomes. However, their efficacy is often limited by off-target toxicity, poor bioavailability, and acquired drug resistance, especially in advanced or recurrent disease [2].

Extracellular vesicles (EVs) are naturally secreted nanoscale particles that mediate intercellular communication by transferring bioactive molecules, such as proteins, lipids, and nucleic acids. They regulate various physiological and pathological processes, including cancer development and progression [3,4,5]. EVs have gained increasing attention as drug delivery platforms for cancer therapy due to their inherent biocompatibility, low immunogenicity, and ease of modification, making them ideal candidates for precision medicine. Recent studies have demonstrated that EVs can effectively deliver chemotherapeutic agents, microRNAs (miRNAs), small interfering RNAs (siRNAs), and CRISPR-Cas9 components to ovarian cancer cells, enhancing therapeutic efficacy while reducing systemic toxicity [6,7,8].

This review highlights recent advances in EV-based therapeutic strategies for ovarian cancer, emphasizing their applications in delivering chemotherapeutic agents, nucleic acid therapeutics, and immunomodulatory molecules. Moreover, it distinguishes itself from the prior literature by presenting a structured and engineering-oriented synthesis of these strategies. Specifically, it offers (1) a detailed examination of advanced targeting approaches—including EV surface modification, donor cell selection, and biomaterial integration; (2) a dedicated section on magnetic nanoparticle-assisted delivery, highlighting its potential as a novel precision targeting modality; and (3) a translational perspective that examines current clinical challenges, integrating multidisciplinary insights from nanotechnology, pharmacology, and cancer biology.

## 2. Biological Characteristics and Therapeutic Potential of EVs

EVs have been traditionally classified into three main subtypes based on size, biogenesis, and function: exosomes, microvesicles (MVs), and apoptotic bodies. Exosomes (30–150 nm) originate from the endosomal system and are released into the extracellular space upon the fusion of multivesicular bodies (MVBs) with the plasma membrane. MVs (100–1000 nm) are formed through the direct outward budding of the plasma membrane. Apoptotic bodies (>1000 nm) are released as cellular debris during programmed cell death [9].

However, the Minimal Information for Studies of Extracellular Vesicles (MISEV) guidelines recommend using more precise terminology based on EV characteristics [10]. Rather than classifying EVs as exosomes, MVs, or apoptotic bodies, MISEV suggests categorizing them as small EVs (sEVs) and medium/large EVs (m/lEVs) when size is the primary defining factor. Alternatively, classification can be based on surface markers, cargo content, or functional properties when such information is available. This nomenclature is more practical, as EVs cannot be reliably separated based on their biogenesis pathways in experimental settings. Since current isolation methods primarily rely on size and density rather than intracellular origin, adopting a classification system based on measurable characteristics improves clarity and reproducibility in EV research [10].

According to the MISEV2023 guidelines, accurate EV characterization requires the integration of multiple complementary techniques [10]. Physical characterization commonly includes methods such as nanoparticle tracking analysis (NTA), dynamic light scattering (DLS), and resistive pulse sensing (RPS), all of which allow for the quantification of EV size distribution and concentration. Morphological analysis is typically conducted using transmission electron microscopy (TEM) or atomic force microscopy (AFM), which provide high-resolution imaging to confirm vesicle structure and size. Molecular profiling involves the identification of EV markers—such as CD9, CD63, CD81, ALIX, and TSG101—using techniques such as Western blotting or flow cytometry. In addition, recent advances in single-particle analysis, such as single-particle interferometric reflectance imaging sensing (SP-IRIS) and super-resolution microscopy, enable the precise detection and characterization of individual EVs at the nanoscale [11,12,13].

One of the most significant advantages of EVs used as drug delivery systems is their natural biocompatibility. Compared to conventional drug delivery systems, such as liposomes or polymeric nanoparticles, EVs exhibit lower immunogenicity and toxicity. Their ability to mimic natural cell-to-cell communication allows them to interact with target tissues in a more physiologically relevant manner, reducing the risk of adverse side effects [14]. Additionally, the use of autologous or patient-derived EVs further minimizes immune rejection, making them a safer option for personalized medicine [15,16,17]. The reduced off-target toxicity of EVs is particularly beneficial in ovarian cancer therapy, where systemic chemotherapy often leads to severe side effects, including nephrotoxicity, neurotoxicity, and myelosuppression [18].

Furthermore, the lipid bilayer of EVs effectively protects encapsulated therapeutic cargo from enzymatic degradation, enhancing stability during circulation and promoting efficient delivery to target tissues. Additionally, EVs exhibit homotypic targeting and self-recognition capabilities, further enabling personalized treatment approaches [19,20,21]. Continued progress in EV engineering, scalable production methods, and clinical validation will be critical for translating EV-based therapies into mainstream ovarian cancer treatment.

## 3. Therapeutic Applications of EVs in Ovarian Cancer

**Figure 1:**
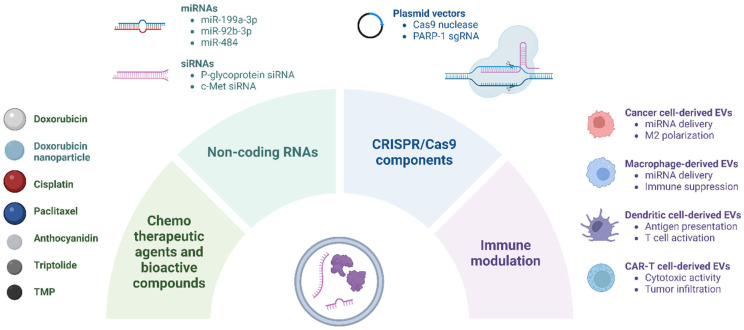
EV-based delivery of therapeutic agents in ovarian cancer. EVs are engineered to encapsulate various therapeutic cargoes, including chemotherapeutic agents, bioactive compounds, nucleic acid therapeutics (e.g., miRNAs, siRNAs, and CRISPR-Cas9 components), and immunomodulatory molecules. CAR, chimeric antigen receptor; PARP-1, poly (ADP-ribose) polymerase-1; sgRNA, single-guide RNA; TMP, tetramethylpyrazine.

This section reviews the current landscape of EV-based therapeutic strategies in ovarian cancer, focusing on their use in delivering chemotherapeutic agents, bioactive compounds, and nucleic acid therapeutics, as well as their role in immune modulation (Figure 1).

### 3.1. Delivery of Chemotherapeutic Agents and Bioactive Compounds

#### 3.1.1. Doxorubicin

EV-based drug delivery has been successfully utilized to transport chemotherapeutic agents to ovarian cancer cells, offering a promising alternative to conventional formulations (Table 1). A notable example is the EV-mediated delivery of doxorubicin, a widely used chemotherapeutic agent. Although effective, the clinical use of doxorubicin is significantly limited by cardiotoxicity, particularly with prolonged exposure. Encapsulating doxorubicin within EVs helps mitigate these adverse effects by reducing non-specific cellular uptake and improving drug pharmacokinetics. Studies have demonstrated that exosomal doxorubicin can increase the therapeutic index, enhancing efficacy while minimizing systemic toxicity [22,23]. A key advantage of exosomal encapsulation is its ability to limit doxorubicin penetration through myocardial endothelial cells, thereby reducing cardiac toxicity. In preclinical tumor models, mice treated with exosomal doxorubicin tolerated higher drug concentrations, resulting in improved anti-tumor effects [22]. In a similar study, exosomal doxorubicin exhibited superior potency compared to both free doxorubicin and liposomal formulations across multiple cancer cell lines and primary cells [23].

Additionally, doxorubicin-loaded EV-mimetic nanovesicles engineered with folic acid demonstrated improved tumor targeting and reduced drug resistance in ovarian cancer [24]. Recently, orange-derived EVs (OEVs) were introduced as novel nanodrug carriers through surface modification with cyclic arginyl-glycyl-aspartic acid (cRGD)-targeted doxorubicin nanoparticles (DN@OEV) [25]. This EV-based delivery system significantly enhanced tumor accumulation and penetration, leading to the more efficient inhibition of ovarian cancer growth. Mechanistically, DN@OEV promoted receptor-mediated endocytosis, enhancing drug delivery by directing drugs preferentially through the early endosome/recycling endosome pathway, which facilitates exocytosis and reduces drug degradation in the lysosomal pathway. This optimized trafficking process increased transcytosis efficiency and improved drug delivery to cancer cells [25].

#### 3.1.2. Cisplatin

Chemoresistance remains a significant challenge in the treatment of ovarian cancer, particularly for platinum-based therapies like cisplatin. Despite its potent cytotoxic effects, the clinical efficacy of cisplatin is often compromised by drug resistance mechanisms and severe systemic toxicities, including nephrotoxicity, neurotoxicity, and myelosuppression [18]. A recent study investigated the feasibility of loading cisplatin into exosomes derived from umbilical cord blood (UCB) macrophages to enhance its therapeutic efficacy against platinum-resistant ovarian cancer [26]. M1 and M2 macrophage-derived exosomes were evaluated as drug carriers, with results demonstrating that M1 exosomes loaded with cisplatin (M1-exoCIS) exhibited significantly greater cytotoxicity in both cisplatin-sensitive (A2780) and cisplatin-resistant (A2780/DDP) ovarian cancer cells.

One of the key mechanisms contributing to cisplatin resistance is endosomal trapping, where cisplatin is sequestered within intracellular compartments, preventing it from reaching its nuclear DNA target. To overcome this limitation, another study investigated the use of milk-derived exosomes for cisplatin delivery in cisplatin-resistant ovarian cancer [27]. The findings demonstrated that cisplatin-loaded milk exosomes enhanced anti-cancer effects compared to free cisplatin in both in vitro and in vivo models. Mechanistically, milk-derived exosomes facilitated cisplatin uptake through clathrin-independent endocytosis and macropinocytosis, bypassing hCtr1-mediated drug transport, which is often downregulated in resistant cancer cells. Notably, milk exosomes also enabled cisplatin to evade endosomal and lysosomal trapping, ensuring more efficient intracellular drug release [27].

#### 3.1.3. Paclitaxel

Paclitaxel is a widely used chemotherapeutic agent that inhibits cancer cell division by stabilizing microtubules and preventing mitosis [32]. A recent study explored the potential of paclitaxel-loaded mesenchymal stem cell (MSC)-derived exosomes for targeted cancer therapy, including SKOV3 ovarian cancer cells. Paclitaxel loading into exosomes was achieved by incubating MSC cultures with a sublethal concentration of paclitaxel for 24 h, followed by exosome isolation. Notably, LC-MS/MS analysis revealed that paclitaxel-loaded MSC-derived exosomes required 7.6 times lower paclitaxel concentrations to achieve the same cytotoxic effects as free paclitaxel [28]. In another study, an exosomal paclitaxel formulation was developed for oral delivery, demonstrating comparable therapeutic efficacy to intraperitoneally administered free paclitaxel. Furthermore, combining exosomal paclitaxel with anthocyanidin-loaded exosomes led to enhanced antitumor activity against A2780 tumor xenografts [29].

#### 3.1.4. Bioactive Compounds

Anthocyanidins have demonstrated anti-cancer properties, including growth inhibition of ovarian cancer cells. However, poor oral bioavailability and stability limit the therapeutic potential of plant bioactives like anthocyanidin. To address these limitations, milk-derived exosomes have been employed as nanocarriers for anthocyanidins. Compared to free anthocyanidins, exosomal formulations exhibited enhanced antiproliferative activity [29]. Triptolide (TP) is a naturally derived diterpenoid with potent anti-cancer properties; however, its clinical application is limited due to poor solubility and organ toxicity [33]. To overcome these challenges, TP-loaded exosomes were developed by co-incubating purified exosomes with triptolide. The resulting TP-loaded exosomes exhibited high encapsulation efficiency, efficient cellular uptake, and effective tumor-targeting capabilities both in vitro and in vivo. Although TP-loaded exosomes provided stronger tumor growth inhibition compared to free TP, they also induced liver and spleen toxicity, emphasizing the need for further optimization [30]. Tetramethylpyrazine (TMP), also known as ligustrazine, is a naturally occurring bioactive alkaloid commonly isolated from the medicinal plant *Ligusticum wallichii*. An exosome-based TMP formulation effectively reversed paclitaxel resistance in A2780T ovarian cancer cells and suppressed tumor growth by downregulating drug resistance proteins [31].

EV-based drug delivery systems represent a promising avenue for overcoming critical barriers in ovarian cancer treatment, notably chemoresistance and systemic toxicities. Future developments in EV engineering, including enhanced targeting strategies and optimized cargo loading methods, could further expand their clinical potential for ovarian cancer patients.

### 3.2. Delivery of Nucleic Acid Therapeutics

EVs can efficiently deliver nucleic acid-based therapeutics—such as non-coding RNAs (ncRNAs) and CRISPR-Cas9 components—to ovarian cancer cells. In one study, exosomes derived from cancer-associated stromal cells were found to contain high levels of miR-21 isomiRNAs. These miRNAs were transferred to ovarian cancer cells, where they promoted chemoresistance by downregulating the apoptosis-related gene APAF1 [34]. While the broad repertoire of nucleic acids naturally encapsulated within EVs has been thoroughly reviewed elsewhere [35,36], this section focuses on studies that employ EVs as biological carriers by loading them with exogenous nucleic acids for therapeutic applications (Table 2).

#### 3.2.1. ncRNAs

miRNAs are small ncRNAs that regulate gene expression at the post-transcriptional level [42]. In one example, the tumor suppressor miR-199a-3p was electroporated into exosomes derived from patient-derived omental fibroblasts. These engineered exosomes elevated miR-199a-3p levels in ovarian cancer cells, suppressed c-Met expression, and inhibited cell proliferation and invasion. In a xenograft mouse model, treatment with miR-199a-3p-loaded exosomes also reduced peritoneal dissemination and downregulated c-Met, extracellular signal-regulated kinase (ERK) phosphorylation, and matrix metallopeptidase 2 (MMP2) expression in tumor tissues [37].

Another promising strategy involves the use of peptide-engineered exosomes overexpressing anti-angiogenic miRNAs. A recent study identified miR-92b-3p as a key regulator of tumor-associated angiogenesis by directly targeting SRY-box transcription factor 4 (SOX4). SKOV3 cells stably overexpressing miR-92b-3p were generated via lentiviral transduction. Exosomes derived from these cells, further modified with tumor-targeting peptides, significantly inhibited endothelial tube formation and cell migration in vitro, as well as angiogenesis and tumor growth in vivo [38]. In another study, EVs loaded with miR-484 effectively reduced chemoresistance in ovarian cancer cells by inhibiting angiogenesis [39].

In addition, siRNAs and plasmid vectors have been successfully delivered using engineered EV systems. One study developed a hybrid delivery platform by combining bovine colostrum-derived exosomes with a polyethyleneimine matrix (EPM), enabling the efficient delivery of nucleic acids. Using this system, K-Ras-targeting siRNA suppressed tumor growth and reduced K-Ras expression, while the plasmid-mediated delivery of wild-type p53 restored its function in p53-null cancer models [43]. In another study, surface-engineered EV-mimetic nanovesicles co-loaded with doxorubicin and P-glycoprotein siRNA effectively reversed drug resistance and enhanced cytotoxicity in multidrug-resistant ovarian cancer cells [24]. Additionally, patient-derived exosomes loaded with c-Met siRNA inhibited ovarian cancer cell proliferation, migration, and invasion and prolonged survival in xenograft models [40].

#### 3.2.2. CRISPR/Cas9 Components

EVs have also been employed as delivery vehicles for CRISPR/Cas9 components. In a recent study, cancer cell-derived exosomes were used as natural carriers for CRISPR/Cas9 plasmid and single-guide RNA (sgRNA) targeting poly (ADP-ribose) polymerase-1 (PARP-1), a critical gene involved in DNA repair. These exosomes demonstrated enhanced tumor tropism and selectively accumulated in SKOV3 ovarian cancer xenografts, enabling efficient in vivo gene editing. The knockdown of PARP-1 via CRISPR/Cas9-loaded exosomes induced apoptosis in ovarian cancer cells and enhanced sensitivity to cisplatin, demonstrating synergistic antitumor effects [41]. Collectively, these studies underscore the versatility of EVs as effective carriers for a wide range of nucleic acid-based therapeutics in ovarian cancer.

### 3.3. Immunomodulation

Immunomodulation plays a crucial role in cancer therapy, with EVs emerging as both a target and a tool for modulating the tumor microenvironment and enhancing anti-tumor immune responses [44]. EVs derived from hypoxic epithelial ovarian cancer (EOC) cells have been shown to transport miRNAs, such as miR-940, into the tumor microenvironment, promoting M2 macrophage polarization and facilitating cancer progression [45].

#### 3.3.1. Macrophage-Derived EVs

Tumor-associated macrophage (TAM)-derived EVs carry miR-29a-3p, which targets forkhead box O3 (FOXO3) and leads to increased programmed death-ligand 1 (PD-L1) expression in ovarian cancer cells. This upregulation of PD-L1 enables cancer cells to evade immune surveillance, thereby driving tumor progression [46]. Furthermore, TAM-derived EVs contribute to EOC progression and metastasis by delivering miR-21-5p and miR-29-3p. These miRNAs disrupt the balance between regulatory T cells (Tregs) and T helper 17 (Th17) cells by inhibiting the signal transducer and activator of the transcription 3 (STAT3) pathway, further promoting an immunosuppressive tumor microenvironment [47]. Additionally, in EOC tissues, EVs derived from PD-L1⁺ TAMs enhance the expression of carnitine palmitoyltransferase 1A (CPT1A) in CD8⁺ T cells by activating the peroxisome proliferator-activated receptor alpha (PPAR-α) pathway. This activation promotes fatty acid oxidation, resulting in the increased apoptosis and exhaustion of CD8⁺ T cells, ultimately facilitating EOC metastasis [48].

#### 3.3.2. Dendritic Cell-Derived EVs

Conversely, EVs derived from dendritic cells (DCs) have been shown to stimulate anti-tumor immunity by presenting tumor antigens and activating T cells. For instance, tumor peptide-pulsed DC-derived exosomes have been demonstrated to prime antigen-specific cytotoxic T lymphocytes (CTLs) in vivo, resulting in effective tumor suppression in murine models in a T cell-dependent manner [49]. A comparative study of lEVs and sEVs secreted by human DCs revealed that both were equally effective in inducing CD4⁺ T-cell activation in vitro [50]. However, under conditions of DC immaturity, functional differences emerged: lEVs favored Th2 cytokine secretion, while sEVs promoted Th1 cytokines such as interferon-gamma (IFN-γ). Interestingly, when DCs matured, these functional differences disappeared, and both lEVs and sEVs were able to induce IFN-γ secretion [50].

Another approach involved engineering dendritic cell-derived nanovesicles (CDNVs) using nitrogen cavitation to replicate DC-derived EV properties [51]. These CDNVs activate antigen-specific T cells via direct and indirect mechanisms and can be produced in large quantities, overcoming production limitations encountered with traditional EVs. Direct T-cell activation occurs through interactions between surface proteins on CDNVs and CD8⁺ T cells. Indirect activation happens when bystander antigen-presenting cells (APCs) internalize CDNVs, subsequently cross-presenting antigenic peptides or acquiring preformed peptide-major histocompatibility complex (pMHC) class I complexes from CDNVs, thereby activating neighboring CD8⁺ T cells [51].

#### 3.3.3. CAR-T Cell-Derived EVs

Chimeric antigen receptor (CAR)-T cell therapy has shown considerable success in hematological malignancies, but its application in solid tumors like ovarian cancer has been limited by complications such as cytokine release syndrome and off-target effects [52]. Recent advancements have explored the use of EVs derived from CAR-T cells as a safer alternative for targeted immunotherapy [53,54]. CAR-T cells release EVs, primarily exosomes, that carry CAR on their surface and exhibit potent anti-tumor activity [55]. These CAR-containing exosomes express high levels of cytotoxic molecules and inhibit tumor growth. Unlike CAR-T cells, CAR exosomes do not express programmed cell death protein 1 (PD-1), making them resistant to recombinant PD-L1 treatment. Additionally, in a preclinical model of cytokine release syndrome, CAR exosomes demonstrated a favorable safety profile compared to CAR-T cell therapy [55]. Notably, unlike their parental cells, CAR exosomes can efficiently infiltrate tumor tissues, even in environments with significant fibrosis, thus improving drug delivery and therapeutic efficacy [54].

#### 3.3.4. Engineering Approaches

Recent advancements in EV engineering have further expanded their potential as immunotherapeutic tools. Genetic engineering approaches have enabled donor cells to produce EVs enriched with immunostimulatory cargo, such as interleukin-12 (IL-12) or co-stimulatory ligands, thereby augmenting anti-tumor immune responses [56,57]. Notably, exosomes engineered to display immune checkpoint modulators (PD-1, OX40L) along with tumor-targeting antibodies (anti-CD3, anti-EGFR) have demonstrated potent T-cell activation and the robust suppression of EGFR-positive tumors in murine models [58]. In addition, the surface engineering of EVs to present MHC molecules loaded with tumor-associated peptides has shown promise in directly priming cytotoxic T lymphocytes, effectively bypassing the need for conventional APCs [59].

In summary, EVs play a dual role in immunomodulation, serving both as mediators of immune evasion within the tumor microenvironment and as potential therapeutic tools for enhancing anti-tumor immunity. While these findings underscore the immense potential of EV-based immunotherapy, further research is required to optimize its clinical application in ovarian cancer.

## 4. Targeting Strategies for EV-Based Delivery

**Figure 2:**
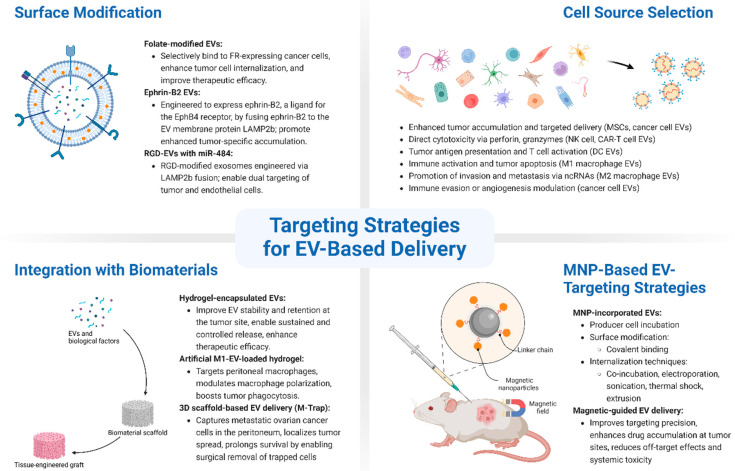
Schematic overview of targeting strategies for EV-based delivery in ovarian cancer. EV targeting can be enhanced through various strategies: (1) surface modification, which involves genetic or chemical engineering of EV membranes to display targeting ligands such as folate, ephrin-B2, or RGD peptides, improving tumor-specific uptake and therapeutic efficacy; (2) cell source selection, where EVs are derived from cells with inherent tumor-tropism (e.g., mesenchymal stem cells, immune cells, or ovarian cancer cells), enabling natural targeting and immune modulation; (3) integration with biomaterials, including hydrogels and 3D-printed scaffolds, which protect EVs, prolong retention at disease sites, and allow for controlled release; and (4) magnetic nanoparticle (MNP)-based strategies, where EVs are loaded with or surface-modified by MNPs to enable magnetic field-guided targeting, enhancing accumulation in tumors and minimizing off-target effects.

The effective targeting of EVs is essential to improve the precision and efficacy of EV-based therapies in cancer treatment. Considering the heterogeneous nature of tumors and the complexity of the tumor microenvironment, various strategies have been developed to enhance the specificity of EVs toward cancer cells (Figure 2).

### 4.1. Surface Modification

Recent advances in the surface engineering of EVs have demonstrated promising potential for targeted delivery. Functionalizing EV surfaces with targeting ligands or antibodies significantly enhances their selective internalization by cancer cells. The genetic modification of EV-producing cells enables the stable incorporation of targeting ligands directly into EV membranes, ensuring robust and consistent expression. For instance, engineering donor cells to express fusion proteins that combine EV membrane proteins with targeting ligands can result in EVs with enhanced specificity for target cells [60]. Chemical modification, such as lipid insertion or click chemistry, allows for the flexible and reversible attachment of ligands or antibodies onto EV surfaces without compromising their biological activity or structural integrity [61].

The folate receptor (FR), a cell surface glycoprotein frequently overexpressed in ovarian cancer and several other cancers, is a particularly attractive target for selective delivery [62]. EVs engineered with folate or FR-targeting conjugates have shown enhanced tumor cell uptake and improved therapeutic efficacy by preferentially binding to FR-expressing cancer cells [63,64,65,66]. For instance, folate-modified exosomes engineered through RNA nanotechnology enabled the efficient delivery of siRNA payloads directly into target cancer cells, effectively bypassing endosomal trapping. These folate-displaying exosomes significantly suppressed tumor growth in preclinical animal models, highlighting their potential for targeted cancer therapy [63].

Similarly, protein and peptide ligands, such as ephrin-B2 and RGD peptides, have been incorporated onto EV surfaces to enhance tumor targeting and improve therapeutic outcomes [39,67]. Alharbi et al. developed EVs engineered to express ephrin-B2, a ligand for the ephrin-B4 receptor, which is frequently overexpressed in ovarian cancer. These ephrin-B2-expressing EVs were isolated from HEK293T cells transfected with a plasmid encoding ephrin-B2 fused to the EV membrane protein lysosomal-associated membrane protein 2 (LAMP2b). The intravenous injection of ephrin-B2-modified EVs into an ID8 ovarian tumor-bearing mouse model resulted in significantly enhanced tumor-specific accumulation [67].

In addition, targeting strategies utilizing RGD peptides—known for their high affinity to integrin receptors, which are often overexpressed in cancer—have shown considerable promise [39]. RGD-modified exosomes were engineered by fusing RGD to LAMP2b, enabling their selective accumulation in ovarian tumor tissue, including both cancer cells and endothelial cells. When loaded with miR-484, these exosomes effectively induced tumor vessel normalization and enhanced chemotherapeutic sensitivity in ovarian cancer xenografts. Mechanistically, miR-484 simultaneously suppressed VEGF-A expression in cancer cells and its receptors in endothelial cells, resulting in increased chemotherapy-induced apoptosis and prolonged survival in treated mice [39].

Surface modification with targeting antibodies is another actively explored strategy for enhancing tumor-specific EV delivery. For instance, exosomes functionalized with anti-EGFR nanobodies demonstrated enhanced uptake by EGFR-expressing tumor cells [68]. Similarly, EVs modified with antibodies targeting carbonic anhydrase 9 (CA9) and glucagon-like peptide-1 receptor (GLP1R) demonstrated cell-specific internalization [69,70]. More recently, a modular platform was developed in which EVs were engineered to display Fc-binding domains on their surface (Fc-EVs), enabling flexible conjugation with various IgG antibodies [71]. This approach allows for the antibody-guided targeting of EVs to specific tissues or tumor cells, depending on the antibody employed. In preclinical models of human epidermal growth factor receptor 2 (HER2)- and PD-L1–positive cancers, antibody-decorated Fc-EVs exhibited significantly improved cellular uptake and tumor accumulation. Furthermore, when loaded with the chemotherapeutic agent doxorubicin, these engineered EVs demonstrated superior anti-tumor efficacy compared to non-targeted controls [71].

### 4.2. Cell Source Selection

The cell type from which EVs are derived plays a crucial role in determining their natural tropism and therapeutic potential [72]. MSCs have emerged as particularly promising sources, primarily due to their inherent tumor-homing capabilities. EVs derived from MSCs exhibit enhanced accumulation at tumor sites, making them attractive vehicles for the targeted delivery of therapeutic cargo to ovarian and other solid tumors [73,74,75,76]. A notable application of MSC-derived EVs is their use as delivery systems for photodynamic therapy (PDT) in the treatment of peritoneal metastases. In this context, MSC-EVs loaded with temoporfin, a photosensitizer used in PDT, were administered to mice bearing peritoneal metastases of colorectal and ovarian origin. The intraperitoneal injection of temoporfin-loaded EVs demonstrated superior tumor targeting and induced potent tumor necrosis, highlighting their potential to enhance the efficacy of PDT [77].

Additionally, immune cell-derived EVs exhibit unique properties for targeted immune modulation, enhancing both direct tumor killing and antitumor immune responses [78,79,80]. Natural killer (NK) cell-derived EVs bind to tumor cells via surface receptors and exert cytotoxic effects by releasing cytotoxic proteins such as perforin, granzymes, and small peptides [81]. T cell-derived EVs display diverse antitumor and immunoregulatory functions. CAR-T cell EVs target tumor cells via CAR recognition and induce cytotoxicity by releasing perforin and granzyme B [82]. DC-derived EVs can present tumor antigens and stimulate specific antitumor immune responses [59]. EVs derived from M1 and M2 macrophages exert opposing effects on tumor progression. M1-derived EVs, enriched with MHC and CD54 molecules, promote T cell activation and tumor apoptosis—effects further supported by their miRNA and long ncRNA (lncRNA) content. In contrast, M2-derived EVs transfer miRNAs and lncRNAs that regulate invasion-related proteins, thereby promoting tumor invasion and metastasis [80].

Ovarian cancer cell-derived EVs possess intrinsic self-homing properties that facilitate selective accumulation within parental tumor tissues. This inherent targeting capability is driven by the molecular signature of EVs, closely mirroring that of their parental cancer cells. Utilizing these self-homing characteristics can enhance the specificity and efficacy of EV-based drug delivery. However, a significant limitation is their dual potential to modulate the tumor microenvironment, possibly promoting angiogenesis or suppressing anti-tumor immune responses, which necessitates careful consideration in therapeutic contexts [83].

### 4.3. Integration with Biomaterials

The integration of EVs with biomaterials has led to innovative strategies for enhancing cancer therapy. One of the major challenges of systemic EV administration is their rapid clearance from circulation, with an estimated plasma half-life of only a few minutes. In addition, a substantial portion of injected EVs accumulates in phagocytes within the liver and spleen, reducing their bioavailability at the target site [84,85,86]. To address this, biomaterials such as hydrogels have been explored as EV carriers to improve delivery efficiency, prolong retention, and enable controlled release at disease sites [87].

Hydrogels—water-swollen, gel-like materials composed of natural or synthetic polymers—have shown great promise as delivery vehicles for EVs. Their porous and degradable structures allow for the sustained release of EVs while protecting them from premature degradation in dynamic biological environments. In addition to their high biocompatibility, hydrogels often possess antibacterial properties and self-healing capabilities, making them an ideal platform for various therapeutic applications. Studies have demonstrated that MSC-derived EVs encapsulated within hydrogels can enhance therapeutic outcomes in both tissue regeneration and cancer treatment [88].

A notable example involves the development of an engineered hydrogel incorporating artificial exosomes derived from genetically modified M1 macrophages. This hydrogel, loaded with the efferocytosis inhibitor MRX-2843, was designed to target peritoneal macrophages for ovarian cancer therapy. Upon X-ray-triggered immunogenic activation, the artificial exosome-based hydrogel modulated macrophage polarization and enhanced tumor cell phagocytosis, effectively bridging innate and adaptive immune responses to improve therapeutic efficacy [89].

However, traditional bulk encapsulation methods for EVs face several challenges, including potential toxicity, undesirable physical alterations, and chemical reactions that can compromise EV stability and function. To address these limitations, advanced microfabrication systems have been developed, offering precise control over EV encapsulation and release profiles. Through micro- and nanofabrication techniques, such as 3D bioprinting, it is possible to design EV-loaded scaffolds with intricate microscale architectures tailored for optimized therapeutic delivery. Encapsulating EVs within 3D scaffolds preserves their structure, stability, and function for extended periods, enabling sustained therapeutic effects. These scaffolds act as protective matrices, shielding EVs from biological environments while ensuring their gradual and controlled release at the disease site [90,91,92].

One particularly innovative strategy involves the creation of M-Trap, a 3D scaffold embedded with exosomes derived from the ascitic fluid of ovarian cancer patients, designed to selectively capture metastatic tumor cells [93]. By integrating patient-derived exosomes into the scaffold, M-Trap establishes a preferential site for metastatic cell adhesion, effectively remodeling the landscape of peritoneal dissemination. In murine models, this system transformed metastatic disease into a localized condition. Moreover, the removal of the scaffold following tumor cell capture further prolonged survival, highlighting the potential of M-Trap as a non-pharmacological, bioengineered approach for controlling cancer metastasis [93].

### 4.4. Magnetic Nanoparticle-Based EV-Targeting Strategies

Recent advancements in nanotechnology have led to the integration of magnetic nanoparticles (MNPs) with EVs as a promising strategy for targeted drug delivery. By utilizing the magnetic properties of nanoparticles, EVs can be guided to specific sites using an external magnetic field, improving their accumulation in targeted tissues while minimizing off-target effects [94].

One approach to magnetic EV targeting involves incubating MNPs, such as superparamagnetic iron oxide nanoparticles (SPIONs), with EV-producing cells. This method results in the incorporation of MNPs into the exosomes during their biogenesis. Studies have demonstrated that these magnetically responsive EVs exhibit enhanced retention at target sites when exposed to an external magnet, enabling precise drug delivery [95]. Another method involves the direct attachment of MNPs to purified EVs by surface modification. Surface attachment techniques use covalent binding molecules, such as transferrin or hydrophobic tails, to functionalize the EV membrane with MNPs [96]. This strategy has been successfully applied to enhance tumor targeting in vivo, where SPION-coated EVs loaded with TNF-α demonstrated potent anti-tumor activity under an external magnetic field [97].

MNPs can also be incorporated into EVs via internalization techniques such as simple incubation, electroporation, sonication, thermal shock, and extrusion [98,99,100,101]. Co-incubation is a widely used method for integrating materials into EVs; however, its loading efficiency is relatively lower compared to other techniques. Electroporation temporarily disrupts the EV membrane, enabling MNPs to enter without significantly compromising membrane integrity. Other methods, such as sonication and thermal shock, facilitate nanoparticle loading by inducing transient membrane openings, though they may pose risks of structural alterations. The extrusion method, which forces EVs through filters with specific pore sizes, has also been explored for cargo loading, though it may modify EV protein composition and potentially alter their biological functions [98,99,100,101].

MNP-enhanced EVs represent an emerging platform for precise and efficient drug delivery. By leveraging the controllable nature of external magnetic fields, these systems offer improved targeting capabilities, increased drug retention at disease sites, and reduced systemic toxicity. Future studies focusing on optimizing loading techniques and enhancing magnetic targeting precision could further expand the clinical potential of these hybrid delivery systems.

## 5. Challenges and Future Perspectives

Despite the promising potential of EV-based therapies, several challenges must be addressed to ensure their successful clinical translation. One of the pressing challenges is the large-scale production of EVs while maintaining consistent quality and functional integrity. Current isolation techniques face limitations in yield and reproducibility. The development of scalable and standardized manufacturing processes is crucial for ensuring clinical-grade EVs with reproducible therapeutic effects.

Another major challenge involves the preservation of EV stability and bioactivity during storage and transport [102,103,104]. Recent studies have demonstrated that preservation strategies—such as cryoprotectant formulations, lyophilization, and controlled freezing protocols—can effectively maintain the structural integrity and functional activity of EVs over extended periods. These approaches have been well documented and systematically summarized in recent articles [105,106,107].

In addition, the pharmacokinetics and biodistribution of EVs following systemic administration remain critical concerns. Rapid clearance by the reticuloendothelial system and potential off-target accumulation pose barriers to effective drug delivery. To address these limitations, various engineering strategies have been explored. For example, the surface modification of EVs with polyethylene glycol (PEG) has been shown to extend circulation time and reduce immunogenicity [108]. Similarly, the overexpression of CD47 on the EV surface can inhibit macrophage-mediated clearance by delivering a “don’t eat me” signal, thereby enhancing systemic persistence [109,110]. Moreover, reducing the abundance of negatively charged phosphatidylserine-derived groups on the EV membrane has been shown to suppress phagocytic uptake by macrophages, further contributing to prolonged circulation and improved stability [111]. Future research should focus on improving these strategies to achieve the sustained and controlled release of therapeutic cargo.

Although EV-based therapeutics generally elicit lower immune responses compared to other delivery systems, safety and immunogenicity remain key concerns, as pro-tumorigenic effects or unintended immune reactions could limit their clinical application [112]. Several studies have reported dose-dependent cytotoxicity, pro-inflammatory responses, and organ-specific accumulation, particularly at high systemic doses or with repeated administration. The heterogeneity in EV origin, purification methods, and cargo loading strategies further contributes to variability in safety profiles. Moreover, engineered EVs—particularly those modified with targeting ligands or loaded with potent therapeutic agents—may induce additional immunogenic or off-target effects [113]. Comprehensive preclinical and clinical studies will be necessary to establish the safety profiles, dosage optimization, and long-term effects of EV-based interventions. Additionally, regulatory hurdles pose a significant barrier, necessitating the establishment of clear guidelines for EV-based drug development and approval. For further details on clinical trials and regulatory aspects of EV-based therapies, readers are referred to recent reviews [114,115,116,117,118].

Advancements in nanotechnology and genetic engineering may further enhance the therapeutic potential of EVs. In recent years, CRISPR-Cas genome editing has emerged as a transformative tool for precise genetic modifications, offering new avenues for ovarian cancer treatment. However, the clinical application of CRISPR-Cas technology is limited by the lack of an efficient and safe delivery vehicle capable of targeting tumor cells. EVs offer a promising solution by protecting CRISPR-Cas components from degradation and enabling targeted gene editing within the tumor microenvironment. Additionally, integrating EV-based CRISPR-Cas delivery with immunotherapy could enhance anti-tumor immunity by knocking out immune-regulatory genes, potentially improving treatment efficacy [119].

Recent discoveries of non-vesicular extracellular particles (NVEPs), such as exomeres and supermeres, present new opportunities for therapeutic applications [120]. Exomeres, which are smaller than traditional EVs (<50 nm), are non-membranous nanovesicles with unique protein and lipid profiles. They were first identified using asymmetric flow field flow fractionation (AF4) [121]. Following the isolation of exomeres, additional centrifugation of the supernatant led to the identification of a new class of EPs, termed supermeres [122]. Even smaller than exomeres, supermeres exhibit distinct biological properties, including the ability to cross the blood–brain barrier. Notably, they have been implicated in drug resistance mechanisms, such as transferring cetuximab resistance between cancer cells, underscoring their potential role in therapy resistance [122]. Tumor-derived EPs may serve as promising targets for therapeutic intervention. Moreover, EPs with self-homing properties could serve as efficient drug delivery vehicles, providing a novel strategy for targeted cancer therapy.

In summary, this review presents recent advances in EV-based therapeutic strategies for ovarian cancer, emphasizing their roles in the targeted delivery of chemotherapeutics, nucleic acid-based agents, and immunomodulatory molecules. Engineering innovations—such as EV surface modification, donor cell selection, and integration with functional biomaterials—have significantly enhanced the specificity, stability, and therapeutic efficacy of EVs. While challenges remain regarding large-scale production, safety, pharmacokinetics, and regulatory approval, continued progress in EV engineering and translational research is expected to accelerate their clinical application. As such, EVs hold considerable potential as next-generation platforms for precision medicine, offering safer and more effective treatment options for ovarian cancer.

## Figures and Tables

**Table 1:** EV-based delivery of chemotherapeutic agents and bioactive compounds.

Cargo	Vehicles	Effects	References
Doxorubicin	Cell-derived exosomes	Reduced cardiotoxicity by limiting doxorubicin penetration into myocardial endothelial cells; enabled higher dosing, resulting in enhanced antitumor efficacy in ovarian cancer models	[22,23]
Doxorubicin	EV mimetic nanovesicles	Reversed multidrug resistance, enhanced tumor targeting, and suppressed tumor growth without adverse effects in drug-resistant ovarian cancer models	[24]
Doxorubicin nanoparticle	Orange-derived EVs	Enhanced tumor accumulation and penetration via transcytosis; inhibited ovarian cancer growth in orthotopic models	[25]
Cisplatin	Macrophage-derived exosomes	Enhanced cisplatin cytotoxicity in both drug-sensitive (A2780) and drug-resistant (A2780/DDP) ovarian cancer cells	[26]
Cisplatin	Milk-derived exosomes	Enhanced anti-cancer efficacy of cisplatin in resistant ovarian cancer cells via exosome-mediated clathrin-independent endocytosis and evasion of endosomal trapping	[27]
Paclitaxel	MSC-derived exosomes	Induced cytotoxicity in ovarian cancer cells in vitro and reduced primary tumor size and distant metastases in vivo using exosomes at lower drug concentrations	[28]
Paclitaxel	Milk-derived exosomes	Enhanced antiproliferative activity in ovarian cancer cells; improved oral bioavailability; enhanced antitumor activity against A2780 tumor xenografts when combined with anthocyanidin-loaded exosomes	[29]
Anthocyanidin	Milk-derived exosomes	Enhanced antitumor activity against A2780 tumor xenografts when combined with paclitaxel-loaded exosomes	[29]
Triptolide	SKOV3-derived exosomes	Inhibited proliferation and induced apoptosis in SKOV3 ovarian cancer cells; enhanced tumor-targeting and antitumor efficacy in vivo; reduced systemic toxicity	[30]
TMP	Cancer cell-derived exosomes	Reversed multidrug resistance in A2780T ovarian cancer cells; enhanced paclitaxel efficacy; downregulated drug resistance–related proteins; induced apoptosis in drug-resistant cells	[31]

DDP, cisplatin; MSC, mesenchymal stem cell; TMP, tetramethylpyrazine.

**Table 2:** EV-based delivery of nucleic acid therapeutics.

Cargo	Vehicles	Effects	Reference
miR-199a-3p	Omental fibroblast-derived exosomes	Inhibition of cell proliferation and invasion; reduction in peritoneal dissemination in a xenograft mouse model	[37]
miR-92b-3p	SKOV3-derived exosomes	Inhibition of endothelial tube formation; suppression of cell migration; reduction in angiogenesis in vivo; decreased tumor growth in vivo	[38]
miR-484	HEK293T-derived exosomes	Downregulation of VEGF-A and its receptors; induction of tumor vessel normalization; enhanced chemotherapy-induced apoptosis; prolonged survival in tumor-bearing mice	[39]
P-glycoprotein siRNA	EV mimetic nanovesicles	Reversal of multidrug resistance in ovarian cancer cells; enhanced cytotoxicity in drug-resistant ovarian cancer models; effective tumor targeting and suppression of tumor growth	[24]
c-Met siRNA	Patient-derived fibroblast exosomes	Inhibition of cancer cell proliferation, migration, and invasion; selective accumulation in peritoneal tumors after intraperitoneal injection; suppression of downstream oncogenic signaling; prolonged survival in xenograft mouse models	[40]
CRISPR/Cas9-targeting PARP-1	SKOV3-derived exosomes	Knockdown of PARP-1 expression; induction of apoptosis in cancer cells; enhanced cisplatin sensitivity; synergistic antitumor effect	[41]

PARP-1, poly (ADP-ribose) polymerase-1; VEGF, vascular endothelial growth factor.
